# Did the COVID-19 quarantine policies applied in Cochabamba, Bolivia mitigated cases successfully? an interrupted time series analysis

**DOI:** 10.1080/16549716.2024.2371184

**Published:** 2024-07-01

**Authors:** Rodrigo K. Arce Cardozo, Osvaldo Fonseca-Rodríguez, Yercin Mamani Ortiz, Miguel San Sebastian, Frida Jonsson

**Affiliations:** a Department of Epidemiology and Global Health, Umea University, Umea, Sweden; b Biomedical and Social Research Institute, “Aurelio Melean” Medical School, San Simon University, Cochabamba, Bolivia

**Keywords:** Pandemic, policy, health service, evaluation, Latin America, time-series

## Abstract

**Background:**

The COVID-19 pandemic prompted varied policy responses globally, with Latin America facing unique challenges. A detailed examination of these policies’ impacts on health systems is crucial, particularly in Bolivia, where information about policy implementation and outcomes is limited.

**Objective:**

To describe the COVID-19 testing trends and evaluate the effects of quarantine measures on these trends in Cochabamba, Bolivia.

**Methods:**

Utilizing COVID-19 testing data from the Cochabamba Department Health Service for the 2020–2022 period. Stratified testing rates in the health system sectors were first estimated followed by an interrupted time series analysis using a quasi-Poisson regression model for assessing the quarantine effects on the mitigation of cases during surge periods.

**Results:**

The public sector reported the larger percentage of tests (65%), followed by the private sector (23%) with almost double as many tests as the public-social security sector (11%). In the time series analysis, a correlation between the implementation of quarantine policies and a decrease in the slope of positive rates of COVID-19 cases was observed compared to periods without or with reduced quarantine policies.

**Conclusion:**

This research underscores the local health system disparities and the effectiveness of stringent quarantine measures in curbing COVID-19 transmission in the Cochabamba region. The findings stress the importance of the measures’ intensity and duration, providing valuable lessons for Bolivia and beyond. As the global community learns from the pandemic, these insights are critical for shaping resilient and effective health policy responses.

## Paper Context

- **Main findings:** The findings highlight the importance of stringent quarantine measures in managing infectious disease outbreaks, offering valuable insights for policymakers worldwide in strategizing effective public health interventions.
- **Added knowledge:** By providing a detailed analysis of testing disparities and quarantine policies’ effectiveness within a specific Latin American context, our research fills a critical gap in understanding their impacts on health system responses and disease control.
- **Global health impact for policy and action:** The findings highlight the importance of stringent quarantine measures in managing infectious disease outbreaks, offering valuable insights for policymakers worldwide in strategizing effective public health interventions.

## Background

While the COVID-19 pandemic has instigated extensive societal and behavioral transformations, economic challenges, heightened discrimination, and health inequalities [1–4], many of these ramifications remain insufficiently examined or addressed. These challenges, and particularly the increase in cases and health system saturation, have exerted substantial pressure on health systems globally, emphasizing the urgency of research in this arena, particularly in low- and middle-income countries (LMICs) [5]. Throughout the pandemic, different periods of case increase or waves emerged, with governments in LMICs implementing diverse interventions to counteract its effects. However, no single measure has proven universally efficacious, underscoring the situation’s complexity [6]. One of the most used strategies for containment, mitigation, and overall case reduction was the implementation of quarantines.

These varied greatly on several levels, including target population, duration, and stringency. Evaluating the impact of these measures in specific contexts is vital to understanding their success or failure, and is necessary to address future emergency responses [7].

Studies show that when comparing Latin America with the rest of the world, the COVID-19 pandemic interventions were either less effective in achieving similar outcomes than other regions or suffered from heterogeneous data quality and availability [8]. Specifically, responses to COVID-19 among countries in Latin America were very diverse and had little to no coordination.

They involved: 1) quarantines (a primary measure of disease control); 2) travel restrictions; 3) social distancing and masking mandates; 4) vaccine utilization; 5) improvements in health system infrastructure e.g. building more hospitals or creating new Intensive Care Units in some cases doubling previous hospital capacity, and 6) economic and social harm reduction initiatives like economic support for families via conditional cash transfers of the Bolivian government [9].

Due to a notable scarcity of published studies on the COVID-19 pandemic response in Latin America in comparison to other regions, numerous crucial aspects remain insufficiently addressed [10]. For instance, the World Bank has highlighted that healthcare systems in Latin America exhibited suboptimal performance compared to the Organization for Economic Cooperation and Development average [11]. When looking at regional differences in outcomes, studies varied extensively regarding the outcomes and predictors used. Latin America reported almost 2.5 times COVID-19 lethality compared to the rest of the world [12]. Notably, the increase in tests per population emerged as a robust negative predictor of mortality, while increases in the gross domestic product exhibited a positive correlation [12,13]. These counterintuitive effects might stem from limited access to tests and other health system resources, coupled with the concurrent underdiagnosis of cases. It is also important to note that in this study, we focus on case mitigation and not primary mortality reduction.

To navigate these complex circumstances and comprehend the impact of interventions or policies implemented in response to the pandemic, numerous strategies have been proposed, employed, and discussed [14]. Exploring the implementation of COVID-19 pandemic policies in Latin America and their respective outcomes is a significant opportunity to understand key strengths and weaknesses in the health system’s emergency response to future known and unknown shocks [15–17]. Analyses of quarantine implementation conducted in some countries in the region, including Colombia, Costa Rica, Peru, Ecuador, Mexico, and Chile, concluded that such measures did not guarantee the curve-flattening effect in all countries [18]. In Brazil, a study showed a reduction in cases when quarantines were implemented in several regions but with diverse success estimates [19]. Other analyses that have also focused on country-specific circumstances and implementation strategies of quarantines in certain cities and regions, have shown heterogeneous results [20,21].

One of the countries in the Latin America region where limited COVID-19-related research has been conducted, is Bolivia. Researchers have so far reported on diverse topics related to the pandemic response. They include low support for healthcare workers during the emergency [22], risk factors for mortality and the role of telehealth in the emergency response [23,24]. While these studies provide an important contribution, there is still a lack of research on the effect of the quarantine policies in Bolivia. To address a critical gap in existing knowledge, this study aimed to describe COVID-19 testing trends in the health system and evaluate the effects of the implemented quarantine measures on the COVID-19 case trends in Cochabamba, Bolivia.

## Methods

### Study setting

Bolivia is located centrally in South America. The country’s population is estimated at 12.08 million in 2022 [25,26]. Geographically and administratively, it’s divided into nine departments. Its geography varies from the rugged Andes Mountains with a highland plateau (Altiplano), and hills, to the lowland plains of the Amazon Basin [26]. It is a LMIC, ranking 118 out of 189 countries and territories on the Human Development Index [27], and the public health system receives only 4% of the national gross domestic product.

Cochabamba is centrally located and one of the nine administrative/geographical departments of Bolivia. In 2022, demographic estimates indicated that about 2.1 million people lived in this department [25]. Administratively, it has 16 provinces and 47 municipalities. The province and municipality of Cercado act as the capital for Cochabamba, and it is estimated to have 856,198 inhabitants. The peripheral urban metropolitan area surrounding Cercado is formed by 4 more municipalities that include Quillacollo, Sacaba, Vinto, and Colcapirhua (with 362,387 inhabitants in total) [25].

The Bolivian Health System is comprised of both public and private healthcare sectors, regulated by the Bolivian Ministry of Health (BMoH) [28]. A universal single-payer system was established in February 2019 as a national insurance serving all uninsured habitants, yet its implementation, efficacy, and population reach are still limited [29,30]. The public sector has two main systems; 1) the previously mentioned public healthcare system comprising hospitals, clinics, health centers, and other public services supported by national, regional, and municipal funds; and 2) the social security work-based healthcare system supported by employee contributions. In this article, both public systems will be addressed as independent sectors given their unique characteristics. The private sector includes insurance companies, prepaid medicine companies and services, traditional medicine providers, and nongovernmental organizations (NGOs) with diverse staffing and infrastructure management and organization. All of them are responsible for enacting national policies and strategies established by the BHoM [30]. The BMoH oversees all health services, formulating strategies, and developing plans and programs at the national level, which are then implemented by the other levels of the organization. They manage the data of the health system using the National System of Health Information (SNIS in Spanish), which organizes and consolidates the data of the different health subsystems [28].

### The COVID-19 strategy in Bolivia

The initial response to the COVID-19 pandemic in Bolivia followed a top-down national model, later transitioning to a more decentralized approach at the department and municipal levels in subsequent stages of the response. The Bolivian COVID-19 strategy was directed by the central government, which delegated the responsibility for implementing measures to departmental and municipal managers, along with other key stakeholders.

The first case of SARS-COV2 was confirmed in Bolivia on 10 March 2020. The day after, on March 11, a national emergency was declared [31], resulting in cancellation of events, closing schools and borders, and implementing a national lockdown [32]. On March 17, the Government implemented a national community quarantine with stay-home orders, and lockdowns, followed by other measures such as border closings, institutional or domiciliary quarantines for individuals with positive tests, and contact tracing [33]. Initially, only one person per household was permitted to venture out for essential purchases during the community quarantine periods, and economic subsidies were provided, coupled with penalties for non-compliance [34,35]. The government then designated reference public hospitals to attend only COVID-19 cases in the country [36,37]. Subsequent measures during the second and third waves introduced a dynamic community quarantine based on case incidence (applied to departments, or cities experiencing high transmission rates). This emphasized remote work and implemented disinfection protocols in work and business venues [38–41].

To this date, the country has had 10 waves or case surges, but several estimations indicate significant case underreporting in the official case numbers [42,43]. The Cochabamba department in Bolivia reported some of the worst national lethality rates (10% in Cochabamba, 6.2% nationally) during the first waves of the pandemic [44]. The official reports show that by 30 December 2022, Bolivia had 1,158,948 positive cases, or 9597 cases for 100,000 population, and attributed total mortality of 185 for 100,000 population. The geographical distribution of the reported cases followed population density, larger in urban settings during the first two years of the pandemic 2020–2022 [45]. These numbers need to be addressed with some caution since research shows underdiagnoses in official country estimates [46].

### Data sources and analysis

Cases of COVID-19 in Cochabamba were reported daily during the pandemic using the COVID-19 testing database of the Cochabamba Department Health Service [47]. Reviewing available BMoH data sources and public media communications, we identified the specific periods in time when quarantines were set in place in the Cochabamba Department. The different periods used for testing patterns description and quarantine effects analysis are shown in Table 1. We define quarantine as a period where community quarantines and stay-at-home orders included one or more weekdays for the entire population of the Cochabamba Department. This was in parallel to lockdowns and individual quarantine recommendations for all people who tested positive, for at least one week to prevent further transmission during the entire observation period.

**Table 1.** Quarantine policy timepoints.

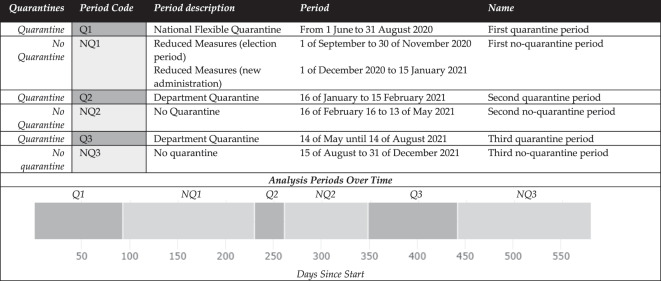

COVID-19 test reporting was mandatory, and in total, 1,048,575 tests were reported to the Department of Health Service, from the beginning of the pandemic on 10 March 2020, until 31 December 2022. The first quarantine lasted from the 21 March until the 31 of May 2020, but the disease was not widely transmitted in the communities in this period, and the testing capacity of the country and department had, at that time, significant limitations. For these reasons, it was excluded from the analysis. Similarly, the last period of 2022, when no formal quarantines were applied, was also excluded. Thus, the analysis included all data points (days) from the beginning of the national quarantine on 1 June 2020, to the last day of no quarantine on 31 December 2021.

We used the test records regardless of the type of test (antigen, serologic, or molecular testing) these were considered equivalent in clinical practice for diagnosing the disease in our observation period. We calculated the outcome as a daily COVID-19 test positive rate and compared it with a 7-day average positive rate (the average of the specific day rate, 3 days rates prior, and 3 days rates after the date) to measure the disease transmission. We decided to use only the daily positive rate since the 7-day period did not significantly reduce the effect of single or multiple-day outliers (holidays, and weekend misreporting) on the trends. Subsequently, an interrupted time series analysis (ITSA) using quasi-Poisson regressions was applied to measure the impact of quarantine versus no quarantine policies implemented on the changes in the daily testing positive rate after the enforcement of the regulations.

Adjusting for seasonality has been described as an important factor to consider when analyzing time trends and waves of cases in the COVID-19 pandemic [48]. After seeing the cyclical behavior of the testing in our data we concluded controlling for season was appropriate. We adjusted our model for seasonality using two methods; first by using a binary dummy variable created for all the days of the months considered as part of the rainy season or winter season and second, by month of the year. Since these two approaches did not change the original estimates, only the original model without seasonal adjustment is reported. The two other seasonal adjusted models are shown in Appendix 1 and 2 (Table A1).

The resulting model can be represented as:

$$\begin{array}{rl} & \mathrm{Yt}\;∼\;\mathrm{quasi}\;-\;\mathrm{Poisson}\left(\mu_{t},\;\theta \right)\\ & \mathrm{Var}\;\;\left(\mathrm{Yt}\right)=\theta \mu \\ & \mathrm{Yt}=\beta 0+\beta 1t+\beta 2\mathrm{Xt}+\beta 3\mathrm{XtTt}+\beta 4\mathrm{Xt}+\beta 5\mathrm{XtTt}+\beta 6\mathrm{Xt}+\beta 7\mathrm{XtTt}+\beta 8\mathrm{Xt}+\beta 9\mathrm{XtTt}+\beta 10\mathrm{Xt}+\beta 11\mathrm{XtTt}+\mathrm{Et} \end{array}$$

Where *Yt* represents the positive rate outcome variable measured at each equally spaced time point (day) *t*, *Tt* is the time since the start of the study, *Xt* is a dummy variable representing the intervention changes, and *XtTt* is an interaction term. *β0* represents the intercept of the outcome variable (daily positive cases/daily total cases), *β*1 is interpreted as the slope in the outcome associated with a daily change for the first observed quarantine period (Q1), *β*2, *β*4, *β*6, *β*8, *β*10 represent the levels of change following the first non-quarantine period (NQ1), second quarantine (Q2), second no-quarantine (NQ2), third quarantine (Q3), and third no-quarantine period (NQ3), respectively. *β*3, *β*5, *β*7, *β*9, *β*11 represent the slope changes following the first non-quarantine (NQ1), second quarantine (Q2), second no-quarantine (NQ2), third quarantine (Q3), and third no-quarantine (NQ3) periods respectively (see the corresponding periods in Table 1).

We also tested for autocorrelation using autocorrelation, residual, and exponential decay plots in our regression with multiple groups to find the appropriate number of lags for the model and tested for delay buffer periods of 3 and 7 days from changes in the policy implemented to be able to take into consideration the average transmission rate of the disease in assessing the effect of the quarantine or no quarantine periods and did not show significant trend differences to the reported trends. All analyses were implemented using RStudio 2022.12.0 to calculate the Incidence Rate Ratio (IRR), and their 95% confidence intervals (CI) for inferential purposes.

## Results

The descriptive analysis of the COVID-19 testing data stratified by sex (women or men), and the different sectors of the department health system are shown in Table 2. During our analysis period, 53.8% of the COVID-19 tests were administered to women, while positive and negative test percentages were quite similar for both sexes. In different health sectors, women also represented a small majority in the population tested, except in the private sector, where the percentage of tests reported was higher among men (55.1%). The public sector reported a larger percentage of tests for the department with 65.0%, while the private sector conducted almost twice as many tests (22.9%) as the public-social security sector (10.6%). NGOs Church sectors in Bolivia directed services aid to specific communities. The mean age of sectors was higher in the private (41.4) and social security sectors (43.5) respectively, with the latter also having a larger proportion of positive tests. Of the total tests performed in the social security subsector, 29.9% were positive compared to 19.6% and 17.8% for the private and public sectors, respectively.

**Table 2.** Number and proportion of COVID-19 testing stratified by sex and healthcare subsector.

Variable	Stratified by Sex		Stratified by Sector				
	Women	Men	Church	NGOs	Private	Public	Social Security
N (% OF TOTAL)	563912 (53.78)	484522 (46.21)	4912 (0.51)	8854 (0.92)	218603 (22.92)	620187 (65.03)	101102 (10.60)
AGE MEAN (SD)	37.53 (18.69)	37.88 (19.10)	33.03 (19.39)	34.85 (17.91)	41.44 (17.76)	35.89 (18.82)	43.51 (19.58)
MEN (% OF TOTAL)	-	-	1920 (39.1)	4366 (49.3)	120340 (55.1)	271956 (43.9)	44895 (44.4)
LABORATORY RESULT (% OF TOTAL) *							
NEGATIVE	404888 (80.5)	354834 (81.1)	4123 (88.0)	6594 (78.7)	168132 (80.4)	463261 (82.2)	47220 (70.1)
POSITIVE	97761 (19.4)	82525 (18.9)	564 (12.0)	1784 (21.3)	40957 (19.6)	100491 (17.8)	20159 (29.9)
LABORATORY METHOD (% OF TOTAL)							
SEROLOGIC METHODS							
ELISA	1555 (0.3)	1348 (0.3)	-	7 (0.1)	2793 (1.3)	9 (0.0)	48 (0.1)
ANTIBODY	2532 (0.5)	2638 (0.6)	-	22 (0.3)	2621 (1.2)	1266 (0.2)	432 (0.5)
MOLECULAR METHODS							
ANTIGEN TESTS	386368 (72.7)	303888 (66.2)	4863 (99.9)	8683 (99.7)	92679 (43.0)	435678 (73.5)	61298 (75.8)
RT- PCR	138466 (26.0)	149103 (32.5)	-	-	117173 (54.3)	151417 (25.5)	18899 (23.4)
GENEEXPER-PCR	2894 (0.5)	2386 (0.5)	-	-	318 (0.1)	4723 (0.8)	239 (0.3)

Access to Polymerase Chain Reaction (PCR) tests was limited, initially by the public sector and later by the social security and private sector. Antigen tests replaced the use of PCR tests given their easier deployment and faster results and were predominately used in the public sector (73.5%) and social security sector (75.8%), but not in the private sector, where PCR molecular tests were more prevalent (54.3%). NGOs and Church services were primarily using antigen tests with close to 99% of all tests conducted.

**Figure 1.**
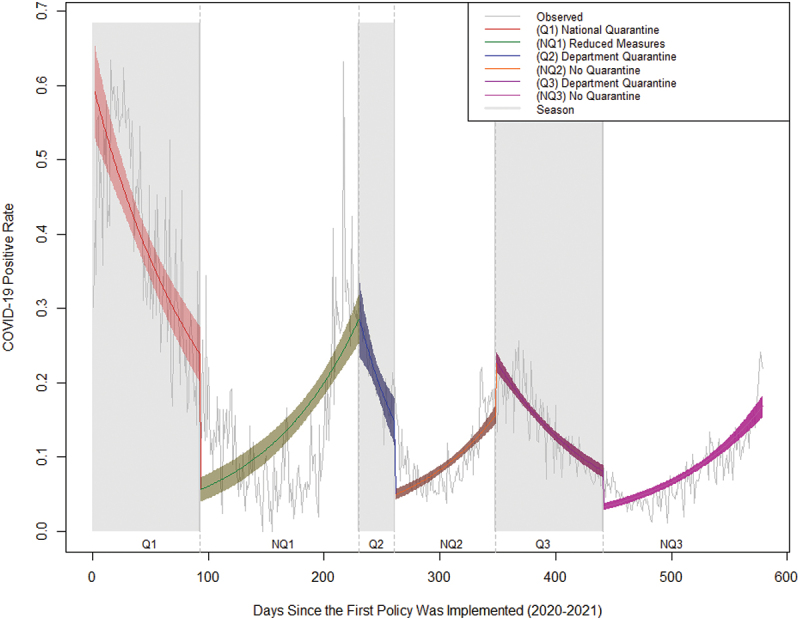
COVID-19 positive incidence rates over time and quarantine period.

Figure 1 displays the trends resulting from the positive incidence rates during the specific quarantine periods and non-quarantine periods. The slope of COVID-19 daily positivity rates varied throughout the different quarantine and non-quarantine periods. During the initial quarantine phase (Q1), there was a negative slope of -0.99% (IRR: 0.99; 95% CI: 0.98–0.99). The relaxation of measures in the first non-quarantine period (NQ1) resulted in a subsequent slope increase of + 2.2% (IRR: 1.02; 95% CI: 1.01–1.02). After the second quarantine period (Q2), a negative slope of -3.3% (IRR: 0.96; 95% CI: 0.95–0.97) was noted. This was followed by a positive slope of + 3.6% (IRR: 1.03; CI: 1.02–1.04) for the subsequent non-quarantine period (NQ2). Upon the enforcement of the third quarantine period (Q3), a negative slope of -2.42% (IRR: 0.97; 95% CI: 0.97–0.97) was evident. Post Q3, there was a positive slope of + 2.34% (IRR: 1.02; 95% CI: 1.02–1.02) during the final non-quarantine period (NQ3).

The results of our model reveal consistent slope patterns in the no quarantine versus quarantine periods with increases and decreases in the COVID-19 positive case rates slopes respectively. The model is shown in Appendix 3.

## Discussion

This study aimed to describe COVID-19 testing trends in the health system and evaluate the effects of the implemented quarantine measures on the COVID-19 case trends in Cochabamba, Bolivia. Our findings provide valuable insights into the dynamic interplay between policies implemented and disease trajectory, offering a compelling case for the efficacy of this stringent measure during periods of heightened transmission risks.

During the 2020–2021 pandemic in the Cochabamba Department, distinct differences emerged among health system sectors regarding test accessibility, positivity rates, and population demographics. Initially, PCR tests were prevalent across sectors, while antigen tests gained prominence later, particularly in public and social security sectors. This shift, possibly due to the simplicity of antigen tests, posed challenges in comparing testing volumes between private and public/social security sectors. PCR tests, requiring trained personnel and laboratory infrastructure, incur higher costs, and are considered more effective in confirming COVID-19 diagnoses. While the private sector had a similar overall percentage of total positive cases as the public sector, the increase in positive cases in the social security sector may be partially explained by the larger proportion of the older population served in that system, as evidenced by an increase in the mean age of this population.

In our main analysis of the quarantine policies applied in Cochabamba, we showed sustained positive and negative slope changes in the case rates, following the implementation of non-quarantine and quarantine periods, respectively. Our results expand on similar studies presented in other countries in the Latin America region [18–20]. A country comparison of a general mandatory quarantine was conducted in Colombia, Peru, and Ecuador also using an interrupted time series analysis to compare pre-and post-policy COVID-19 cases and deaths due to COVID-19 [18]. The implementation of the quarantine did not show a curve-flattening effect in Ecuador and Peru but did so in Colombia. The authors attributed this difference to the reduced capacity to carry out the quarantine measures by the population in Peru and Ecuador. The authors additionally implied a possible difference in resources in Peru and Ecuador compared to Colombia as an explanatory factor of these differences.

In a different study of four regions in Brazil [19], and in synchrony with our results, the authors found that the number of new cases and deaths had a positive trend (increasing) before the quarantines while showing a statistically significant negative trend (decrease) after the quarantines were implemented. However, the authors recognized several weaknesses, including data inconsistencies, testing volume increases during the post-intervention period, and possible random and systematic measurement errors that might have affected their estimates.

Another study conducted in three major cities in Colombia (Bogota, Medellin, and Cali) examined the implementation of quarantine policies employing a differential conditional quarantine approach [20]. In this study, authors found reductions (decreasing trend) in confirmed COVID-19 case numbers per 1,000 inhabitants when the policies were applied, but similarly to other studies, they could not account for testing volume variations, and possible differential testing access and underreporting. Furthermore, the different outcomes or variables used in these studies add complexity to a direct comparison with ours. Many studies analyzing the COVID-19 outcomes in different settings have used mortality metrics, we consider the testing data used in our study a more immediate proxy to the mitigation effects of the quarantines implemented.

The difference in our findings compared to those from other countries in the region may be explained by the stringency of the quarantine in Bolivia. A report from the Inter-American Bank exploring inter-regional variations of the Latin America Region showed that quarantine policies in Bolivia were not only one of the most prolonged in the region, only behind Argentina and Honduras, but also that the intervention was more stringent compared to other countries in 2020 [49]. This is an exploratory conclusion, that could be strengthened with further studies that include stringency metrics between the different contexts being compared.

### Methodological considerations

One notable limitation of our study lies in the introduction of possible measurement errors. This could be due to our model’s inability to incorporate changes associated with variations in testing preferences or testing volume, repeated tests, and possible testing underreporting over time. Even though the testing report was mandatory, and the data includes all reported tests, this may have impacted the reported trends. Similarly, the absence of adjustments for COVID-19 variant-derived transmission or the vaccine introduction could have created alterations in disease transmission patterns (more aggressive transmission of certain circulating variants) or symptom severity reduction and subsequent less testing procurement of the population across the observed periods, representing a gap in our analysis.

Another limitation of our data is the possible difference in testing patterns of rural versus urban health services. In this study, both settings were seen as aggregated since the quarantines were applied nationally or regionally, and we lacked a measure that could identify how well the policies were applied in rural settings, where less control could be expected.

We also recognize the possibility of unknown period misclassification that may have been created by delimiting the periods of this analysis from different sources (newspapers and government documents) since a trustful measure of policy implementation was not available.

Finally, without measures of stringency or reduced mobility in our data, we were unable to control for this in our analysis. We acknowledge that this may have affected our estimates.

## Conclusion

While our study has shown the positive impacts of the quarantine policy interventions on COVID-19 transmission rates in Cochabamba, it also underscores the complexity of pandemic response strategies. Acknowledging the limitations and delving into the nuances of policy effectiveness are critical steps toward refining future emergency mitigation approaches and preparedness. As the global community continues to grapple with the ever-evolving landscape of the COVID-19 pandemic and seeks to prepare for forthcoming crises, continued research is pivotal in LMICs to strengthen the local capacity to conduct rigorous analyses and to use evidence-based policies to manage future emergencies or crisis.

Analyzing the socio-economic and demographic factors that intersect with policy effectiveness could provide valuable insights into vulnerable populations disproportionately affected by the pandemic. Future research could extend our analysis by delving into the nuanced impacts of specific policy components. Examining the differential effects of travel restrictions, social distancing mandates, and mask mandates could provide a granular understanding of their individual contributions to disease containment.

Additionally, exploring the psychological and societal factors influencing public adherence to these policies could elucidate the behavioral dynamics shaping pandemic responses. It is important to note at this point that stringer quarantine measures, in other contexts, have been associated with negative effects on population health in multiple pathways, and many of these are still being explored. Future implementation of such policies should consider social and economic impacts as well as unintended effects on population health.

Research using qualitative methods could capture the experiences and perceptions of individuals affected by COVID-19 in Latin America more generally and Bolivia. This could enrich knowledge with provide more in-depth understanding. Future research could also explore the implications of different variants or vaccine utilization on the observed trends, providing a nuanced insight into the evolving viral landscape.

## Data Availability

Daily count data used in this study is available upon request.

## Appendix 2. Quasipoisson model exponentiated coefficients with and without seasonal adjustment for COVID-19 positive rate

**Table A1.** Quasipoisson model exponentiated coefficients with and without seasonal adjustment.

	Model 1			Model 2 (Season Dummy)			Model 3 (Season Dummy Spline)			Model 4 (Month Dummy)		
Predictors	Log-Mean	CI	p	Log-Mean	CI	p	Log-Mean	CI	p	Log-Mean	CI	P
Baseline (Intercept, Q1)	-0.51	-0.62 – -0.40	<0.001	-0.97	-1.09 – -0.85	<0.001	218.19	184.96–251.30	<0.001	-0.21	-0.37 – -0.05	0.009
Quarantine Slope Change (Q1)	-0.01	-0.01 – -0.01	<0.001	-0.01	-0.01 – -0.01	<0.001	-0.01	-0.01 – -0.01	<0.001	-0.01	-0.01 – -0.01	<0.001
No-quarantine Change (NQ1)	-1.45	-1.79 – -1.13	<0.001	-0.82	-1.12 – -0.53	<0.001	-0.82	-1.12 – -0.53	<0.001	-1.06	-1.41 – -0.74	<0.001
No-quarantine Slope (NQ1)	0.02	0.02–0.03	<0.001	0.02	0.01–0.02	<0.001	0.02	0.01–0.02	<0.001	0.02	0.01–0.02	<0.001
Quarantine Change (Q2)	0.02	-0.20–0.24	0.866	0.07	-0.12–0.25	0.477	0.07	-0.12–0.25	0.477	-0.12	-0.33–0.10	0.287
Quarantine Slope (Q2)	-0.03	-0.05 – -0.02	<0.001	-0.03	-0.04 – -0.02	<0.001	-0.03	-0.04 – -0.02	<0.001	-0.03	-0.04 – -0.01	<0.001
No-quarantine Change (NQ2)	-1.10	-1.36 – -0.85	<0.001	-0.57	-0.79 – -0.34	<0.001	-0.57	-0.79 – -0.34	<0.001	-1.12	-1.37 – -0.87	<0.001
No-quarantine Slope (NQ2)	0.03	0.02–0.05	<0.001	0.03	0.02–0.04	<0.001	0.03	0.02–0.04	<0.001	0.03	0.02–0.05	<0.001
Quarantine Change (Q3)	0.39	0.30–0.49	<0.001	0.25	0.17–0.34	<0.001	0.25	0.17–0.34	<0.001	0.39	0.29–0.48	<0.001
Quarantine Slope (Q3)	-0.02	-0.03 – -0.02	<0.001	-0.02	-0.02 – -0.02	<0.001	-0.02	-0.02 – -0.02	<0.001	-0.02	-0.03 – -0.02	<0.001
No-quarantine Change (NQ3)	-0.91	-1.08 – -0.73	<0.001	-0.42	-0.58 – -0.27	<0.001	-0.42	-0.58 – -0.27	<0.001	-0.91	-1.08 – -0.74	<0.001
No-quarantine Slope (NQ3)	0.02	0.02–0.03	<0.001	0.02	0.02–0.02	<0.001	0.02	0.02–0.02	<0.001	0.02	0.02–0.02	<0.001
Season Dummy Variable				0.46	0.39–0.53	<0.001						
Season Spline							-277.14	-319.11 – -235.03	<0.001			
Month Dummy Variable										-0.05	-0.07 – -0.03	<0.001
Observations	579											
R^2^ Nagelkerke	1.000											

## Appendix 3. Quasipoisson model incidence rate ratios of COVID-19 positive rate

**Table:**

Model 1	COVID -19 Positive Rate	
Predictors	Incidence Rate Ratios	CI
Baseline (Intercept, Q1)	0.603	0.540–0.672
Quarantine Slope Change (Q1)	0.990	0.988–0.993
No-quarantine Change (NQ1)	0.234	0.167–0.324
No-quarantine Slope (NQ1)	1.022	1.018–1.026
Quarantine Change (Q2)	1.019	0.817–1.266
Quarantine Slope (Q2)	0.967	0.956–0.978
No-quarantine Change (NQ2)	0.331	0.257–0.429
No-quarantine Slope (NQ2)	1.036	1.024–1.048
Quarantine Change (Q3)	1.484	1.347–1.636
Quarantine Slope(Q3)	0.976	0.973–0.978
No-quarantine Change (NQ3)	0.404	0.340–0.480
No-quarantine Slope (NQ3)	1.023	1.021–1.026
Days of observación	579	
